# It’s Not You, It’s Me: A Review of Individual Differences in Visuospatial Perspective Taking

**DOI:** 10.1177/17456916221094545

**Published:** 2022-08-22

**Authors:** Steven Samuel, Geoff G. Cole, Madeline J. Eacott

**Affiliations:** 1 Department of Psychology, University of Plymouth; 2 Department of Psychology, University of Essex

**Keywords:** perspective taking, vision, theory of mind

## Abstract

Visuospatial perspective taking (VSPT) concerns the ability to understand something about the visual relationship between an agent or observation point on the one hand and a target or scene on the other. Despite its importance to a wide variety of other abilities, from communication to navigation, and decades of research, there is as yet no theory of VSPT. Indeed, the heterogeneity of results from different (and sometimes the same) VSPT tasks point to a complex picture suggestive of multiple VSPT strategies, individual differences in performance, and context-specific factors that together have a bearing on both the efficiency and accuracy of outcomes. In this article, we review the evidence in search of patterns in the data. We found a number of predictors of VSPT performance but also a number of gaps in understanding that suggest useful pathways for future research and, possibly, a theory (or theories) of VSPT. Overall, this review makes the case for understanding VSPT by better understanding the perspective taker rather than the target agents or their perception.

Successful visuospatial perspective taking (VSPT) concerns the ability of an individual to understand something about the visual relationship between an *agent* or observation point on the one hand and a target or scene on the other. For example, *perspective takers* might judge whether someone can see the salt shaker before they ask them to pass it (e.g., it is not hidden behind a menu), gauge what the view of the stage might be before booking theater seats online, or rapidly assess the blind spots of another driver. VSPT is considered vital to humans’ ability to interact and socialize with others (Brown-Schmidt et al., 2008; Clark & Brennan, 1991) and has been the subject of research and theorizing since at least the time of Piaget (Piaget & Inhelder, 1956). Nevertheless, there is currently no formal theory or model of VSPT (Cole & Millett, 2019; Cole et al., 2020). This is possibly due to the evidence for heterogeneity both in terms of how people take perspectives and how well they do so (Bukowski & Samson, 2017). An important step forward would thus be to extract any patterns from these individual differences.

Here, we review the evidence for individual differences in strategy selection and performance in explicit (i.e., conscious) VSPT. The article is divided into three sections. In the first, we examine whether the means by which a perspective taker opts to solve a VSPT problem (i.e., their strategy choice) is predictable by factors such as gender, culture, schizotypy, and autistic traits. This section also examines some external influences on strategy choice (i.e., direct instructions and the nature of the task). In the second section, we describe and discuss results of studies showing that perspective takers tend to be egocentric and that this affects not only processing efficiency but also accuracy. In the third section, we summarize the evidence for other individual differences in VSPT performance that occur cumulatively with or irrespective of strategy choice and egocentric biases.

Our central argument that follows from this review is that the perspective taker shapes the agent’s perspective, not the other way around. This is evidenced by how perspective takers’ unique experiences affect the nature and efficiency of the attributions they make to others. This approach emphasizes the understanding of individual differences and how these flexibly interact with the variety of VSPT tasks at the same time that it de-emphasizes the role of the visual experiences that one is attempting to understand. What we gain from this understanding is the ability to begin to predict both how and how well people tackle different VSPT problems.

## Factors Influencing Strategy Selection

VSPT problems are usually about visibility (e.g., Is the target object visible to the agent?) or appearance (e.g., Does the target object appear the same to the agent as it does to you?). These problem types correspond to the classic Level 1/Level 2 distinction (Flavell et al., 1981; Masangkay et al., 1974). In experiments that examine Level 1 problems, participants are often given tasks that require them to interpret instructions according to an agent’s more restricted viewpoint. For example, in the director task the instruction to select the “top cup” in an array might require the participants to select the middle cup from their own perspective if the real top cup is not in the agent’s field of view (e.g., Apperly et al., 2010; Keysar et al., 2003). Tasks that examine the ability to understand relative appearance, on the other hand, typically require participants to judge what an object looks like to another person when it is mutually visible to both the perspective taker and the agent, such as whether a digit looks like a “6” or a “9” (e.g., Samuel, Cole, & Eacott, 2020; Surtees et al., 2012, 2013b), how objects that are closer to an agent loom larger (Samuel, Hagspiel, Eacott, & Cole, 2021), and how colors appear different to agents if they view objects through color filters (Samuel, Frohnwieser, et al., 2020).

It is a characteristic of VSPT that there are multiple and distinct cognitive strategies that individuals have at their disposal to solve a problem (Gardner et al., 2013; Pearson et al., 2013). These strategies include but are unlikely to be limited to line-of-sight drawing (Michelon & Zacks, 2006), imagining oneself physically relocated in other spaces (Kessler & Rutherford, 2010), mentally rotating scenes until they align with one’s own perspective (Wraga et al., 2000), understanding the spatial relationships between target objects and occluders (Santiesteban et al., 2015), and reversing left/right mappings for agents opposite (Yu & Zacks, 2017). In essence, by “VSPT strategy,” we therefore mean the procedure that the perspective taker goes through to formulate a response. Because the efficiency and accuracy of responses are in part predicated on this choice, if researchers wish to understand and make predictions about how VSPT works, then they need to understand the factors that lead an individual to select one strategy over another. At the same time, researchers need to understand what the consequences of different strategies would be for different people.

For relatively simple visibility questions (i.e., Level 1 VSPT), it can suffice to “draw a line” from the agent’s eyes and conclude a target is seen if the line is uninterrupted. This strategy was elegantly demonstrated by Michelon and Zacks (2006), who found that adults took longer to judge whether a doll “saw” a target the further away the object was from the doll (attributed to the time taken to “draw” this line). Note that for this type of problem it is not important to know what the object looks like to the agent, only that it is visible at all. More complex Level 1 VSPT questions, such as arrays with multiple objects and occlusions, and Level 2 (appearance) problems would require different strategies. For example, in the director task, the participant is faced with a complex arrangement of multiple objects and barriers to process. It has been argued that under such circumstances participants might prioritize an understanding of the relationship between the objects and the barriers themselves—object-centered spatial coding—rather than attempt to understand what the agent actually “sees” (Heyes, 2014; Santiesteban et al., 2015). Note that object-centered spatial coding, like line of sight, still obviates the need for any holistic representation of the agent’s visual experience. By a representation, psychologists and philosophers typically mean something in one person’s mind (the perspective taker in this case) that “stands for” something in the world (the agent’s perspective, in this case). Even some Level 2 problems, usually associated with a need to generate precisely such a holistic representation (Lurz, 2009), do not always require more than a skeletal notion of another’s perspective. For example, for left/right judgments from the perspective of an agent directly opposite (facing), Yu and Zacks (2017) pointed out that the simple heuristic of reversing left/right mappings could be applied.

These are just some of the strategies involved in VSPT. To illustrate how knowledge of individual differences in strategy choice is currently insufficient, versions of the director task in which the director is removed and participants are instead instructed to ignore items in front of occluders (making the task rule-based and nonsocial) generate both poorer and better performance relative to the original, director-led version (Apperly et al., 2010; Dumontheil, Apperly, & Blakemore, 2010; Dumontheil, Küster, et al., 2010). A potential explanation for this variability is individual differences in strategy preferences, with some participants performing the director-absent version as if it were a perspective-taking task and others performing the director-present version using object-centered spatial coding. Likewise, one might surmise that a perspective taker who knows that a “6” and a “9” look like each other when upside down can apply this knowledge to VSPT problems based solely on the agent’s location relative to the digit. In contrast, an individual approaching this problem for the first time might select a more cognitively demanding strategy, such as mentally rotating the digit.

There are some areas in which the understanding of strategy selection is better. This is the case for the influence of instructions. It is a frequent finding that simply asking a participant to use one particular strategy can produce evidence of that strategy’s use even if the strategy in question is suboptimal and the participant would have no reason to suspect that noncompliance would be detectable. This is most evident in research assessing embodied VSPT and mental-rotation strategies. We discuss this in more detail below because it provides a good test case not only for the influence of external instruction but also for a number of influences on strategy selection more generally.

### Case study: embodiment versus mental rotation

Embodied perspective taking and array rotation are two well-known and cognitively distinct strategies for Level 2 VSPT. The former concerns imagining oneself in the location that provides the desired view and then making a judgment from this quasi-egocentric perspective. This process is known as “perspective transformation,” “viewer rotation,” or simply “embodiment” (e.g., Kessler & Thomson, 2010; Wraga et al., 2000; Zacks & Tversky, 2005). Evidence for this process comes from impaired performance when participants’ bodies are rotated or restricted such that the shortest path to an imagined location is more difficult to attain (e.g., Kessler & Thomson, 2010; Surtees et al., 2013b; Yu & Zacks, 2017) and from erroneous manual responses consistent with perspectives just imagined rather than one’s actual location (Samuel, Legg, et al., 2020). A striking example comes from a study in which participants performed a visual perspective task in virtual reality while sitting in a chair that could be rotated by the experimenters. Deroualle et al. (2015) found that the participants were faster to adopt an alternative perspective if the chair rotated in the same direction as the movement required to “reach” that new viewpoint. Because participants were wearing a virtual-reality headset, the feeling of motion alone (vestibular sensations) in the absence of visual feedback was enough to influence performance.

The other strategy is to mentally rotate the target object or scene instead, variably known as “array rotation,” “object rotation,” and “mental rotation” (Wraga et al., 2000, 2003; Zacks & Tversky, 2005). This is usually considered analogous to the ability to mentally rotate objects more generally (Shepard & Metzler, 1971).

Although both embodied and mental-rotation processes should culminate in identical outcomes, the distinction has been demonstrated both neurologically (Schurz et al., 2013; Zacks et al., 2003) and behaviorally. For example, Kessler and Thomson (2010) asked participants to make judgments about other visual perspectives or mentally rotate objects and observed an influence of congruent or incongruent body posture only with the former. In the aforementioned vestibular-sensations study by Deroualle et al. (2015), there was no effect of the rotating chair if the task was not to imagine other viewpoints but to mentally rotate objects instead.

The selection of one strategy or another is determined in part by the nature of the VSPT task itself. For example, Zacks and Michelon (2005) reported that adults prefer to mentally rotate objects for same/different judgments but prefer embodiment for left/right judgments. Judgments about objects can also elicit a different strategy from judgments about bodies (e.g., Amorim et al., 2006; Muto & Nagai, 2020). Strategy selection is also influenced by overt instructions, to the extent that a more suboptimal approach can be elicited simply by request (e.g., Presson, 1982; Wraga et al., 2000; Zacks & Tversky, 2005).

Instructions and target types are clearly external pressures on VSPT strategy rather than individual differences, but there is also evidence that strategy selection can be predicted by what is known about perspective takers. In healthy adults, responses to many VSPT tasks are usually achieved more quickly using embodiment than array rotation (Amorim & Stucchi, 1997; May, 2004; Zacks & Tversky, 2005), suggesting this might be the preferred way in which people solve VSPT tasks. There is evidence that this preference might be stronger in women. Kessler and Wang (2012) found that the effect of manipulating adults’ body posture while making left/right judgments from alternative perspectives was stronger for women than for men, an effect the researchers attributed to the tendency for women to be greater “empathizers” and men greater “systemizers” (Baron-Cohen & Wheelwright, 2004), with empathizers attempting a “deeper” embodiment (see also Gronholm et al., 2012).

Another example of both preference and flexibility comes from a study that compared adults with high and low schizotypy. “Schizotypy” refers to a multidimensional construct that is thought to underlie schizophrenia and is expressed in the personality of people with and without clinical diagnosis (Kwapil & Barrantes-Vidal, 2015; Wong & Raine, 2020). Schizophrenia is typically associated with atypical theory of mind (ToM), the ability to understand other people’s unobservable mental states, such as their beliefs (Frith, 2004; Harrington et al., 2005; Lee et al., 2004) and perspective taking (Langdon & Coltheart, 1999). In a VSPT task, Langdon and Coltheart (2001) presented 40 adults with four colored blocks arranged in a square on a stand and asked them questions about the appearance of the array from different angles. The questions were either item questions (i.e., “What color block would be to the FRONT and RIGHT?”) or appearance questions, which involved judging whether a picture matched what the array should look like after the transformation was imagined. Participants were instructed to imagine either that the stand that the blocks were on was rotated (array rotation) or that they themselves were “rotated” around the table (viewer rotation/embodiment). The participants were also divided into a “high-” or “low-schizotypy” group on the basis of a median split of responses to a questionnaire designed to measure schizotypal traits. Results showed that both groups performed similarly on item questions, but on appearance questions the high-schizotypal group performed more slowly than the low-schizotypal group in the viewer-rotation condition. In addition, the high-schizotypal group alone found viewer rotation more difficult than array rotation and performed faster than the low-schizotypal group on array-rotation trials. The researchers explained the performance of the high-schizotypy participants in terms of a reduced ability to simulate and select between multiple “first-person” viewpoints of a fixed reality, compensated for with an enhanced ability to imagine alternative realities relative to the self. That this effect was found in healthy adults is particularly striking (the impairment in the embodied condition has also been found in patients with schizophrenia: Langdon et al., 2001).

Like schizophrenia and schizotypy, autism has also been associated with atypicality or deficits in the ability to reason about others’ mental states (e.g., Baron-Cohen, 1995; Frith, 2001; Leslie & Thaiss, 1992), although recent scholarship and research suggest the matter is more complex than previously thought and may itself reflect a particular case of individual differences and intersubjective perspective taking (e.g., Brewer et al., 2016; Milton, 2012; Williams, 2021). Here, we concern ourselves with evidence for differences in VSPT strategies specifically. In a Level 2 VSPT study comparing autistic children and typically developing control subjects, Pearson et al. (2016) found that both groups were as good at judging what a three-dimensional figure looked like from different angles when they were asked to imagine themselves in a different location around the figure as when they were asked to imagine what someone else would see from similar locations. However, they also found that the autistic children’s performance was related to a mental-rotation task, but the typically developing control subjects’ performance was related to a body-matching task, suggesting that the two groups nevertheless adopted different strategies (see also Hamilton et al., 2009; Kessler et al., 2014; Kessler & Wang, 2012).

The case of embodiment versus mental rotation demonstrates the roles of both internal and external factors in VSPT strategy selection: Individuals tend to come to a VSPT problem with a preference for a particular strategy, a preference that is partly predictable by individual differences such as gender, culture, and clinical or subclinical traits. Nevertheless, these preferences are readily undermined by formal instructions. These findings favor a flexible, plural, and context-specific approach to VSPT, which can begin to be understood better by learning more about the perspective takers at their center.

### Folk optics as wildcard strategies

Embodiment and rotation are not the only means by which people choose to tackle VSPT problems. Because VSPT problems are usually accompanied by an instruction or desire to understand aspects of an agent’s visual experience, some people apply their understanding of vision to a problem. However, there is evidence that many people hold quite erroneous theories about how vision really works. For example, in the Venus effect, an observer sees agents looking into a mirror and also sees the agents’ reflections. Observers tend to believe that the agents see their reflection in the mirror as they do despite the agents and mirror not being along the participant’s line of sight (Bertamini et al., 2003; Bertamini & Soranzo, 2018). This effect occurs in approximately 75% of adults (Bertamini et al., 2003), and it is an illusion exploited by art and film to avoid revealing the camera or artist in scenes in which performers look into mirrors. Such effects have been attributed to naive or folk optics (i.e., folk beliefs about how vision works). These beliefs vary from person to person and can be inconsistent with accepted science and even people’s own declarative knowledge (Croucher et al., 2002; Samuel, Hagspiel, Cole, & Eacott, 2021). Different theories and heuristics related to visual reasoning, such as imagining top-down geometric viewpoints or applying past experience of moving through similar scenes, lead to different responses to the same problems depending on whom is asked (Bertamini & Soranzo, 2018; Croucher et al., 2002).

**Fig. 1.**
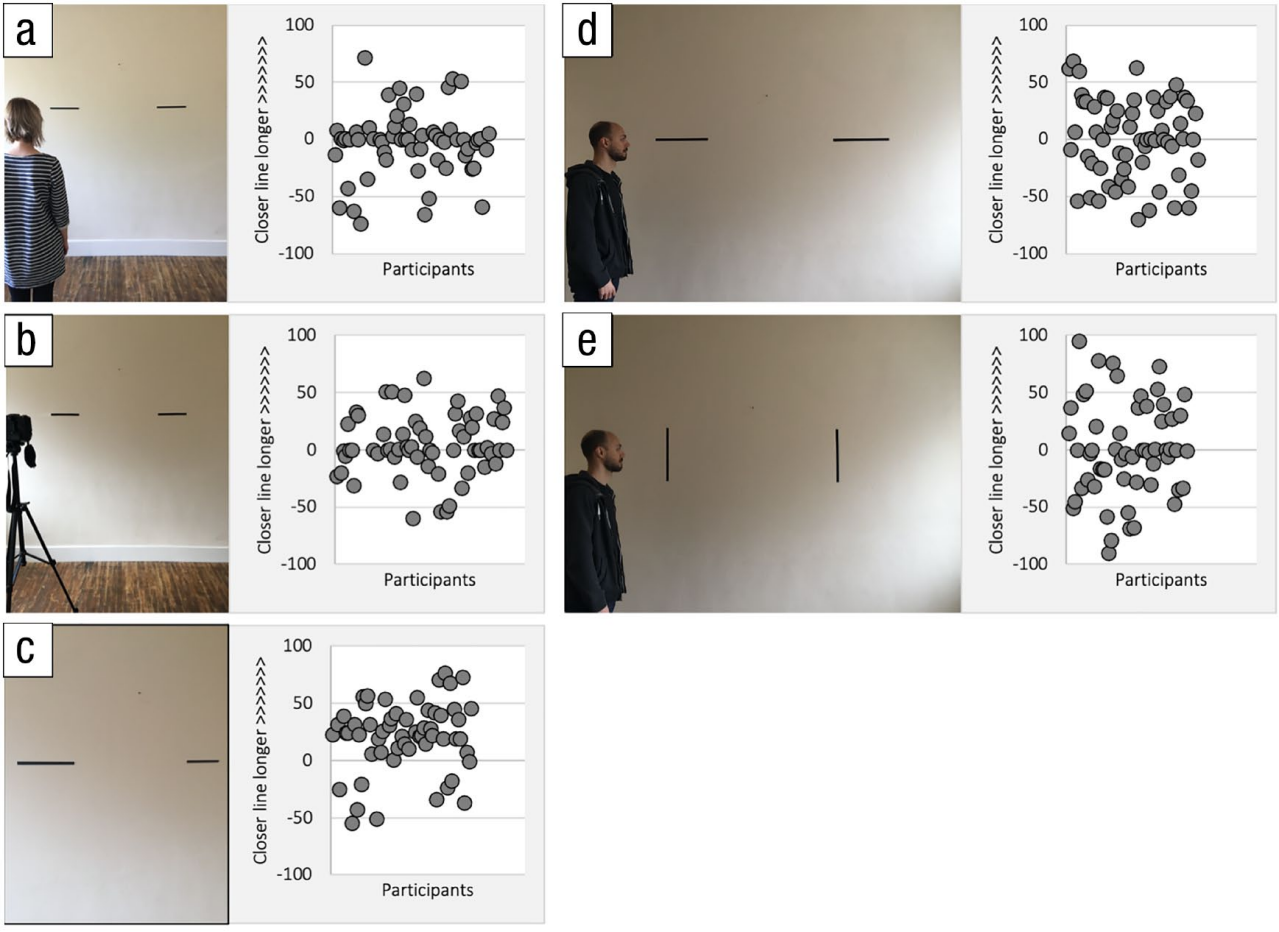
Example stimuli from Samuel, Hagspiel, Eacott, and Cole (2021) and spread of results. Participants responded by judging the length of each line using a slider. Here each dot in the scatterplot represents the judgment of a single participant; points above the zero mark indicate the closer line was judged longer (closer line judgment minus further line judgment). Note the spread of responses suggesting approximately as many participants erroneously judged the further line to appear longer except when they saw a photo taken from the agent’s location (c).

When applied to VSPT, folk optics could lead to unexpected and inaccurate responses. However, to date there is little research linking folk optics to VSPT. In a recent study by Samuel, Hagspiel, Eacott, and Cole (2021), adults were presented with an image of an agent looking at two lines on a wall (Fig. 1a) and were asked to indicate how long each line appeared to that person. The lines were identical, but the closer line to the agent appeared visually longer, as the photo taken from the agent’s location in Figure 1c shows. It was made clear to participants that the agent knew that the two lines were of the same length in reality and that the aim was to judge visual appearance. Results showed no evidence that participants could successfully take the agent’s perspective of the lines. This result persisted when the agent was replaced by a camera and participants asked how long the lines would appear in a photo, eliminating the possibility that they were correcting for the agent’s knowledge that the lines were identical (Fig. 1b). The effect also persisted when the disparity with the participant’s view was made more salient and the lines were turned 90° to ensure that participants’ difficulty was not based on problems extending length into depth (Figs. 1d and 1e). However, participants had little trouble judging the closer line to appear longer when they saw the aforementioned photo (Fig. 1c). Surprisingly, the data from these experiments showed that participants were not simply judging the lines to appear the same length; about as many participants erroneously judged the further line to appear longer as shorter (those data points below the zero midline in the scatterplots to the right of each image). One explanation could be that these participants applied folk optics; in this case, the erroneous belief that visual processing compensates for stretch into depth by enlarging more distant objects. Different beliefs about how vision works help explain why presenting multiple participants with the same stimuli will generate a range of responses, even opposite responses, that cannot be attributed to or predicted by the agent’s real perspective. Until more is known about naive optics and how these relate to VSPT, it will be difficult to predict the outcome of VSPT tasks that rely heavily on them.

### Summary and predictions

**Fig. 2.**
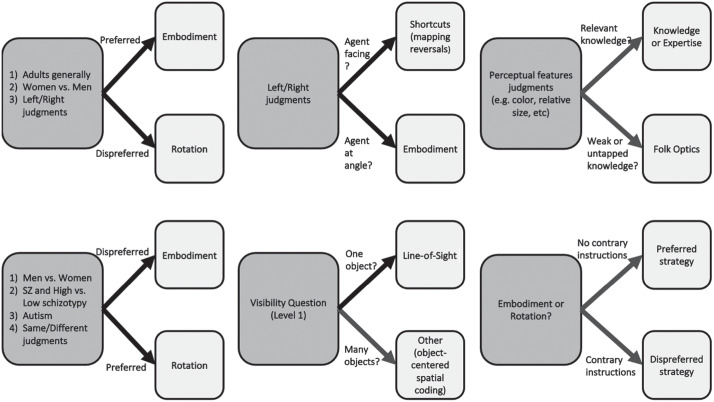
Suggested preferred-strategy-selection pathways. SZ = schizophrenia.

We have seen how there are various means by which VSPT tasks can be approached. Strategy selection will depend in part on the nature of the problem; if it is a visibility judgment then the chances that a line of sight process will be selected are increased, but if a left/right judgment is required then an embodied process or reversal of spatial mappings should be favored. Individual differences also predict VSPT strategy. Expertise should influence strategy selection because the perspective taker should feel more confident using a more practiced strategy. For example, a tour guide who is used to reversing left/right mapping for listeners should be more likely to adopt this heuristic for left/right judgments than people without such experience, who might instead go the “longer” way around by using embodiment or mental rotation. We have discussed the evidence that women appear biased toward embodiment but that autism and schizophrenia (and their nonclinical counterparts) tend to bias people away from it. We have discussed how strategy selection can influence the accuracy of outcomes negatively if an individual is encouraged to use a personally suboptimal strategy. Folk optics are also strategies and represent a “wild-card” influence on strategy selection given that they might lead an individual to bypass entirely the “classic” strategies of embodiment and rotation. They can also lead to wildly inaccurate responses. Figure 2 summarizes these predictions.

## It’s Not You, It’s Me: Egocentric Bias

Egocentric bias is the tendency for one’s own privileged knowledge to interfere when attempting to be objective about other people’s experiences (e.g., Birch & Bloom, 2004; Keysar et al., 2000; Ross et al., 1977). For example, people overextend to others their opinions (Mullen et al., 1985; Ross et al., 1977), their personality and behavior (Dunning & Hayes, 1996; Van Boven et al., 2005), and their valuations of objects (Van Boven et al., 2000). People even show egocentric bias when judging others’ sensations of pleasantness or unpleasantness (Silani et al., 2013) and drive states such as thirst and discomfort (Nordgren et al., 2011; Van Boven & Loewenstein, 2003). As a minimum, egocentric biases in perspective taking demonstrate the difficulty that perspective takers have in making truly objective judgments about other agents’ experiences.

Egocentric bias has also been demonstrated in VSPT. For example, in the director task described earlier, participants are required to select objects from an array according to instructions from a director whose view is limited by occluders. Participants are often slower to select an object from an agent’s perspective if there is a competitor object that matches the instruction but nevertheless only they can see (e.g., Epley et al., 2004; Keysar et al., 2003; Wu & Keysar, 2007). In some versions of the dot-perspective task, in which participants are instructed to verify how many dots an avatar sees, adults are slower on trials in which the avatar sees a different number from themselves (e.g., Qureshi et al., 2010; Samson et al., 2010; Santiesteban et al., 2014).

The continuing relevance of the self-perspective is also evident in tasks with more complex stimuli. Surtees et al. (2012) found that children and adults were slower to verify which number an agent saw if their own perspective made it look different, such as an upside-down “6” appearing to be a “9,” than if the number was identical regardless of perspective, such as an “8” (see also Surtees et al., 2016). A recent study from our own lab (Samuel, Cole, & Eacott, 2020) found not one but two different types of interference from the self-perspective. Participants needed to locate a target digit (a “6” or a “4”) in a grid from the perspective of an avatar. The target was always upright from the avatar’s perspective, and the entire scene was always presented in top-down view such that if the avatar was at the top of the grid, the digits would be upside down for the participant. We found the classic “pull” of an egocentrically correct distractor; participants were slower to select a “6” from the avatar’s perspective if there was an alternative “6” in the array from their own viewpoint (e.g., Keysar et al., 2003). In addition, we also found that responses were slower when participants had to select an upside-down “6” than an upside-down “4.” This suggested that participants were reluctant to select a target that is not merely an ambiguous shape but another number. Participants’ knowledge thus influences performance in not one but two ways: the ability to ignore what is egocentrically correct and the ability to select a target with an alternative identity to the instruction. Critically, each of these factors concern things only the perspective taker sees, yet they affect responses that are meant to reflect the perspective of another agent.

The results described above come from response-time paradigms, which index processing efficiency but tell researchers little when it comes to thinking about how they actually represent other perspectives (Cole & Millett, 2019). However, perspective takers’ egocentric bias also seems to extend to their understanding of what the agent sees. For example, in the director task, participants not only experience interference from objects only they can see but also select them (Apperly et al., 2010; Epley et al., 2004; Keysar et al., 2003), even when under no time pressure (Legg et al., 2017). This seems to suggest that they attribute their own knowledge to others. However, there is an alternative explanation. Perspective takers may fail on such tasks not because they imagine the agent sees what they see but because they fail to engage in perspective taking at all (effectively performing the task as if no other agent existed). The case for misattribution would be stronger if researchers could be more certain that the perspective taker was trying to pay attention to the agent’s perspective when responding. This is the case in a series of studies by Wardlow (2013) and others, in which participants gave rather than received the instructions. She found that descriptions of a target were often qualified with a contrasting adjective such as “small” or “large” when the participant could see a different-sized competitor but the listener could see only one item matching the description (see also Damen et al., 2019; Wardlow Lane & Ferreira, 2008; Wardlow Lane & Liersch, 2012). The important point about these results is that perspective takers are less likely to be ignoring agents when they are describing objects for them and when they are real persons directly in front of them rather than a computer avatar. These errors are thus more likely to be perspective misattributions (i.e., believing that the other agent sees what the participant sees when this is untrue) rather than neglecting to consider the agent’s point of view at all. Just as response times in VSPT tasks can be intentionally “dialed up” by presenting perspective takers with egocentric distractors and misleading targets (Samuel, Cole, & Eacott, 2020), the tendency to egocentrically *misrepresent* other perspectives can be enhanced by presenting conflicts such as contrasting pairs that are visible only to the perspective taker (Wardlow, 2013).

In sum, egocentric bias is a degrader both of efficiency and objectivity in VSPT. Egocentric bias also makes sense because perspective takers attribute perspectives and cannot “take” them—it follows that these attributions will be colored by one’s own knowledge and perceptions. We can therefore hypothesize that a perspective taker’s understanding of other visual perspectives is predictable by the knowledge and perceptions of the perspective taker; the greater the correspondence between these and the perspective of the agent, the more accurate their perspective estimates are likely to be. It also follows that a better informed perspective taker should produce more accurate responses than a less well-informed perspective taker. For example, someone knowledgeable in color mixing should be better able to judge that a yellow object perceived by an agent through a blue filter will appear green than someone without this knowledge.

## Individual Differences in Performance

We have seen how strategy selection can have an impact on VSPT performance and how egocentric bias can degrade it. There is also evidence for individual differences in VSPT accuracy more broadly. However, the understanding of these variables is currently limited, and what is known often comes from developmental studies and belief reasoning (ToM) rather than adults or VSPT. Nevertheless, what evidence there is converges on the distinct likelihood that such factors also influence VSPT performance.

### Culture

Evidence for cultural variation in ToM performance comes largely from developmental studies. This work suggests that the relationship is a complex one concerning not only accuracy but also the stages at which different components of ToM emerge (e.g., Liu et al., 2008; Wellman, 2017; Wellman et al., 2001). Influences of culture demonstrate flexibility and cognitive penetrability and are problematic for nativistic theories of social cognition (Lillard, 1998; Wellman, 2018).

It is unclear how much evidence from ToM/belief reasoning can tell researchers about VSPT specifically. We are aware of only three studies that explicitly related culture to VSPT. Wu and Keysar (2007) reported that Chinese nationals were more sensitive to other agents’ perspectives than Americans on the director task both in terms of their fixation patterns and their behavioral responses. They attributed this finding to the Chinese participants’ interdependent, collectivistic culture that promotes consideration of nonegocentric views (see also Wu et al., 2013). In other work assessing both visibility judgments and left/right judgments from an avatar’s perspective, East Asian (Chinese) adults were again better (i.e., faster) than their Western counterparts (Kessler et al., 2014). Like Wu and colleagues, the authors speculated that the reason for this was the more other-centered culture of the East Asian group. However, using a computerized director task, Wang et al. (2019) found no meaningful differences between a group of Taiwanese (interdependent) and British (independent) adults. There are a number of methodological differences between these studies that might explain this discrepancy, but only with further research on culture in adult VSPT is a clearer picture likely to emerge.

### Bilingualism

An alternative or perhaps complementary explanation for the better performance of the Asian samples in the studies by Wu and Keysar (2007) and Kessler et al. (2014) is that all or part of the sample were recruited from English-language universities and therefore bilingual. Bilingual children have sometimes been found to outperform monolinguals on ToM tasks (Kovács, 2009), including VSPT (for reviews, see Rubio-Fernández, 2017; Schroeder, 2018). For example, Goetz (2003) found that English-Mandarin bilingual pre- schoolers outperformed their monolingual peers when it came to judging whether a picture of a turtle showed it on its feet or on its back from the perspective of someone sitting opposite them, although at a second time of testing one week later the monolinguals had already caught up. Evidence from adult populations is both more limited and more equivocal. Better performance by bilinguals has been reported on a false-belief task (Rubio-Fernández & Glucksberg, 2012) but not in a referential communication task (Ryskin et al., 2014) or director task (Samuel et al., 2016). More research is clearly needed and also an understanding of why bilingualism may offer a potential advantage in VSPT. One explanation is that the management of two languages enhances executive function (EF; e.g., Bialystok, 2017), but this claim has proven controversial, and a number of large-scale studies, reviews, and meta-analyses have failed to find supporting evidence (e.g., De Bruin et al., 2015; Paap et al., 2015; Samuel et al., 2018). An alternative account is that bilingualism teaches an individual that concepts and their labels are not inextricably linked (i.e., greater metalinguistic awareness; see Leopold, 1971; Vygotsky, 1962) and, by extension, views of the world are more person-specific than might be assumed by monolinguals (for a discussion of metalinguistic awareness and its relation to perspective taking, see Doherty & Perner, 2020; see also Rubio-Fernández, 2017). The two accounts differ in emphasis. The former explains enhanced performance through practice with domain-general processes, but the latter through enhanced metarepresentation and appreciation of the subjectivity of experiences. Overall, it appears that an influence of bilingualism on VSPT performance is a possibility that requires further research before firmer conclusions can be drawn.

### Social class

The evidence for the potential for socioeconomic status to influence VSPT performance is preliminary. Dietze and Knowles (2021) recently reported that adults who self-reported belonging to a “lower” category on a social class scale (range included “poor, working class, middle class, upper-middle class, or upper class”) performed better on a director task than adults who judged themselves higher, a finding they speculate could be due to individuals lower on the scale believing that others are more relevant to their experience.

### EF

With the exception of spontaneous perspective taking (e.g., Qureshi et al., 2010; Samson et al., 2010; Ward et al., 2019), most scholars concur that VSPT is an effortful process (e.g., Apperly & Butterfill, 2009; Apperly et al., 2010; Lin et al., 2010). EFs are domain-general abilities, such as inhibition, which regulate performance on a broad range of tasks (e.g., Miyake & Friedman, 2012). A substantial body of research in ToM, particularly but not exclusively in development, has demonstrated that more efficient inhibitory control correlates with better performance on perspective-taking tasks (e.g., Brown-Schmidt, 2009; Carlson et al., 2015; Sabbagh et al., 2006; Samson et al., 2005). These results are usually attributed to the ability to successfully suppress prepotent and perspective-inappropriate (i.e., egocentric) information when considering others’ points of view (Brown-Schmidt, 2009; Carlson et al., 2004). Inhibition has also been related to the ability to flexibly switch between one’s own beliefs and another’s (Bradford et al., 2015).

Similar relationships have often been found between EF and VSPT specifically. For example, in tasks in which adults describe objects for another person in the presence of a competitor in privileged ground, a greater use of redundant adjectives is associated with lower working memory capacity (Wardlow, 2013) and inhibitory control (Long et al., 2018; Wardlow, 2013). In a director task, Lin et al. (2010) found that adults with better working memory spent less time between first fixating and then selecting targets and less time considering a competitor in privileged ground. They also found these participants made fewer errors overall. In a further variant of the director task, Samuel et al. (2019) found that adults were faster on trials from an agent who shared their perspective (self-perspective trials) than trials from an agent who did not (other-perspective trials) but that this egocentric advantage disappeared on self-perspective trials that immediately followed an other-perspective trial. The researchers attributed this result to the likelihood that participants applied inhibition to the egocentric perspective to perform other-perspective trials and that this inhibition had not decayed sufficiently before the following self-perspective trial (see also Chiu et al., 2020). In a similar task, children’s ability to ignore competitors in privileged ground was related to a measure of their inhibitory control (Nilsen & Graham, 2009).

The influence of EF on VSPT performance allows researchers to make predictions about participant *x*’s performance on VSPT task *y* according to *x*’s EF performance (*z*). It also demonstrates again the utility of exploring VSPT beginning from the perspective taker. However, EF is a complex and multifaceted suite of abilities, and how each component might relate to VSPT (and indeed different VSPT problems) is not well understood. For example, sometimes individual differences in EF do *not* translate into performance on VSPT or sometimes do so only in limited ways. In a Level 2 VSPT task, Samuel, Cole, and Eacott (2020) found no evidence that adults’ performance on a test of inhibition (the Simon task: Simon & Rudell, 1967) correlated with either the ability to ignore egocentrically correct competitors or the ability to select a visually misleading but nevertheless correct target. Qureshi et al. (2020) found that adults’ performance on two different VSPT tasks was predicted by *different* measures of EF and, moreover, that performance on these tasks did not correlate with each other, although this discrepancy might have been due to strong methodological differences such as the presence of both self- and other-perspective trials in one task (dot-perspective task) but only other-perspective trials in the other (director task). Overall, the balance of the evidence suggests some role for individual differences in EF as predictors of VSPT performance, but precisely how and how much remain to be learned.

### Summary and predictions

Overall, the evidence for individual differences in VSPT performance is preliminary but suggests a number of potential relationships. There is some support for enhanced performance as a function of a more other-centered rather than individualistic culture and bilingualism relative to monolingualism, although in some cases these different predictors may have been conflated. To date, a single study has suggested that lower social class predicts better performance on a director task. Research assessing a potential relationship between EF and VSPT is more plentiful but continues to paint a complex and sometimes erratic picture. An interesting question for any of these variables is precisely how and at what stage in processing they might influence VSPT performance. For instance, would coming from a more other-centered culture mean that the balance of “self” and “other” perspective information is already tilted in favor of the latter, or does it mean that egocentricity is overcome more easily but at a later stage, just before response?

### Conclusions and limitations

This review makes the case for understanding VSPT by better understanding the perspective taker rather than the agent or the agent’s perception. There is an analogy here with theorizing that has occurred in a quite different field. In his book on language evolution, *The Talking Ape: How Language Evolved*, Robbins Burling (2005) pointed out that people ask other people if they can *speak* a language, not if they can *understand* it. He argued that this emphasizes the wrong end of the equation; any innovation in language production would have died with its creator if the ability to comprehend the new language had not evolved earlier. Language evolution is thus the story of the evolution of comprehension. In the same sense, perspective taking is an ability whose scope and limitations are set not by the agent but by the perspective taker.

The influence of individual differences in VSPT appears to run counter to recent claims that privilege the agent’s perspective over the perspective taker’s (e.g., Quesque et al., 2018) because individual differences should have little impact if this were the case. In recent years, evidence has emerged that people might take other people’s visual perspectives spontaneously (i.e., without conscious effort or awareness). Initially, it was speculated that only a limited amount of information could be gleaned in this manner, such as what an agent sees but not necessarily how that appeared (Samson et al., 2010). More recently, it has been argued that spontaneous VSPT extends to Level 2 VSPT and can also provide perspective takers with information complementary to their own perspective such that objects can be recognized more efficiently if others are better placed to identify them (Ward et al., 2020). This review is concerned with explicit VSPT, and thus it does not speak directly to the debate around spontaneous VSPT (e.g., Apperly & Butterfill, 2009; Cole & Millett, 2019; Santiesteban et al., 2014; Ward et al., 2019). However, we take it as uncontroversial that in any form of VSPT a perspective taker attributes a perspective to an agent and not the other way around (Cole et al., 2020; Cole & Millett, 2019). As we have shown throughout this review, judgments about agents’ perspectives can vary widely among perspective takers even when the agent’s perspective is held constant throughout. Thus, the agent’s perspective is unlikely to be a major factor in VSPT except as a yardstick by which to measure the accuracy of perspective takers’ attributions.

The plurality of VSPT strategies also rules out, to our minds, the possibility that people can represent other visual perspectives veridically (i.e., in a quasi-perceptual or image-like form). This is the claim made by Ward and colleagues (Ward et al., 2019, 2020), who also argued that such representations are generated spontaneously. If this claim were correct, then there would need to be an explanation as to why people do not use these presumably highly accurate representations all the time, rather than these explicit and sometimes suboptimal strategies, and why people are often inaccurate in their perspective attributions (Samuel, Hagspiel, Eacott, & Cole, 2021).

There is often a meaningful distinction between the way in which people solve VSPT problems and how they deal with nonperceptual questions about others’ beliefs, knowledge, emotions, and so on. For example, people cannot usually make judgments about others’ emotions using line of sight, although what someone is attending to might be helpful to infer emotional states (and others’ emotional states might boost attention to others’ lines of sight, e.g., in fearful faces; e.g., Tipples, 2006). For this reason, we do not extend our conclusions to ToM. Of course, this is despite the fact that much research in ToM actually employs tasks that pivot on other agents’ visual perspectives but only in the limited sense that “what an agent sees is what an agent knows about.” For example, the classic false-belief task and its variants rely on agents not seeing the target object move, rendering their belief false (e.g., Keysar et al., 2003; Krupenye et al., 2016; Wimmer & Perner, 1983). Even assessments of Level 2 problems typically pivot on manipulating knowledge and how this then interacts with appearance judgments, such as knowing that what looks like a rock is in fact a sponge (Flavell et al., 1981; Masangkay et al., 1974). Most strategies involved in VSPT do not relate to such questions, or at least not directly. However, one exception could be embodied VSPT, for which there is evidence that it can influence the perspective taker’s sensitivity also to nonperceptual perspectives. Work by Erle and colleagues has found that even making simple left/right judgments from other agents’ visual perspective can enhance feelings of similarity with the agents, boost sensitivity to their mental states (Erle & Topolinski, 2017), and even facilitate liking of and trust in them (Erle et al., 2018). This would be consistent with the speculation by some scholars that VSPT may have been an evolutionary prerequisite for other forms of psychological perspective taking (Kessler & Thomson, 2010).

Throughout this article, we have talked about VSPT rather than only visual perspective taking (VPT) or spatial perspective taking (SPT). This is in large part because we feel a broader definition is presently better placed to capture information that could be crucial, at least until such time that any differences become clear. In VSPT research, “visual” has traditionally been used to describe tasks in which participants are believed to process what another agent perceives, and “spatial” or “visuospatial” is used to describe tasks in which judgments are made about where objects are in relation to another agent (Erle & Topolinski, 2017; Kessler & Thomson, 2010; Surtees et al., 2013a). However, the line is frequently blurred. For example, Kessler and Thomson (2010) used the term “spatial” perspective taking but nevertheless argued explicitly for a role of perception such that “SPT essentially comprises an emulation of the sensory consequences (visual and proprioceptive) of a mental rotation of the self” (p. 84). It has also been shown that visual- and spatial-perspective tasks rely on overlapping mechanisms (Surtees et al., 2013a). In our view, issues around terminology and definitions are understandable given the absence of a theory of visual perspective taking, in particular a theory concerning how people *represent* others’ perspectives, if that is what occurs (Cole et al., 2020; Cole & Millett, 2019; Samuel, Hagspiel, Eacott, & Cole, 2021).

The question of representation is also important for understanding what constitutes success in VSPT and, by extension, what to measure individual differences against. Traditionally, success is measured in terms of consistency with the agent’s viewpoint. However, it is sometimes possible to achieve success on VSPT tasks, even Level 2 tasks, without considering perspective at all. For example, we recently conducted a study in which participants took the perspective of an agent opposite them such that when participants saw a “6,” they should respond that the agent saw a “9” (and vice versa). However, this led some participants (12%–21%) to the error of concluding that when they saw “69,” the agent must have seen “96.” This likely arose from a strategy of “number flipping” whereby the numbers “6” and “9” were inverted and the result of this taken to equate to the agent’s perspective (Samuel et al., 2022). This approach, which we term “stimulus-centered” rather than “agent-centered” could also have led to the correct responses on the single-digit trials.

There are many other questions for which there are presently no firm answers, and this review sheds light on some of these factors but also on the gaps for which knowledge is insufficient. Our review has not been exhaustive; we have not covered the emerging evidence for individual differences in VSPT performance as a function of arguably more temporary psychological states, such as subclinical depression (Erle et al., 2019), and we have focused primarily on a group of the most common VSPT tasks, such as the director task and dot-perspective task. One question that we have not tackled here is whether individuals are aware (or can be made aware) of the strategy they adopt; for example, we know that external factors such as instructions influence strategy selection, which suggests that participants have some conscious control over how they approach VSPT problems, but might some individuals have more or less control over selection, and if so, what might be the explanation? Most pressingly in our view is the need to understand better who selects which VSPT strategies and why. Currently, information is limited to a scattering of studies contrasting vast demographic variables such as “Asian” versus “Western” culture. Overall, the research reviewed here points to VSPT as a flexible and context-specific suite of abilities rather than a one-size-fits-all process or an innate or modular system.

## Transparency

*Action Editor:* Laura A. King

*Editor:* Laura A. King
